# 

**Published:** 1909-10-23

**Authors:** 


### Appointments.

Mr. J. S. Edkins reappointed lecturer on general anatomy and physiology at St. Bartholomew's Hospital.

Mr. A. E. Thomas, Medical Officer of Health of Finsbury, in the place of Dr. C. Porter, resigned.

Dr. Beverley, Medical Officer to the Newbold Urban District Council.

Dr. R. A. Cleveland, who succeeds the late Dr. F. C. Heidenstam, C.M.G., as Chief Medical Officer, Cyprus, has served under the Colonial Office for nearly nineteen years, having been first appointed Medical Officer under the Government of the Leeward Islands, as well as under the Government of the Windward Islands. His services in Cyprus began in 1894, when he was appointed District Medical Officer at Larnaca. He has acted on several occasions as Chief Medical Officer of Cyprus, and was for some years the British Member of the Municipal Commission of the town of Nicosia.

### BOOKS RECEIVED.

Cassell and Co., Ltd.

"Diseases of Stomach." By S. H. Habershon, M.A., M.D., F.R.C.P.

Stevens and Haynes.

"Forensic Chemistry" and "Chemical Science." By W. Jago.

### THE GRADUATES' MEDICAL SCHOOLS.

DIARY FOR THE WEEK, OCTOBER 25 to 30.

### NORTH-EAST LONDON POST-GRADUATE COLLEGE, Prince of Wales's General Hospital, Tottenham, N.

**At 4.30 p.m.—October 26**, Dr. G. N. Meachen, **Demonstration of Selected Skin Cases.**

**October 28**, Mr. H. W. Carson, **Perforated Gastric Ulcer.**

### LONDON SCHOOL OF CLINICAL MEDICINE, Seamen's Hospital, Greenwich, S.E.

**At 2.30 p.m.—October 28**, Dr. Rankin, **Three Recent Cases of Pleurisy.**

**At 3.15 p.m.—October 29**, Mr. McGavin, **Some Points in the Surgical Treatment of Chronic Intestinal Obstruction.**

**At 3.30 p.m.—October 27**, Mr. Cargill, **Some Frequent Mistakes in Ophthalmic Diagnosis.**

### NATIONAL HOSPITAL FOR THE PARALYSED AND EPILEPTIC, Queen Sq., Bloomsbury, W.C.

**At 3.30 p.m.—October 26**, Dr. Batten, **Myatonia Atrophica.**

**October 29**, Dr. Batten, **Myatonia Congenita and its Relation to the Myopathics.**

### THE POST-GRADUATE COLLEGE, West London Hospital, Hammersmith, W.

**At 10 a.m.—October 25** and **28**, Surgical Registrar, **Demonstration.**

**October 29**, Medical Registrar, **Demonstration.**

**At 12 noon.—October 28**, Dr. Bernstein, **Pathological Demonstration.**

**At 12.15 p.m.—October 26**, Dr. Pritchard, **Practical Medicine.**

**October 27** and **30**, Dr. Grainger Stewart, **Practical Medicine.**

**At 5 p.m.—October 25**, Mr. Baldwin, **Practical Surgery, I.**

**October 26**, Dr. Pritchard, **Clinical Pathology.**

**October 27,**

**October 28**, Dr. Davis, **Common Diseases of the Nasal Cavities and Pharynx.**

**October 29**, Dr. Grainger Stewart, **Peripheral Neuritis.**

### MEDICAL GRADUATES' COLLEGE AND POLYCLINIC, 22 Chenies Street, W.C.

**At 4 p.m.—October 25**, Mr. T. P. Beddoes, **Clinique (Skin).**

**At 5.15 p.m.**—Dr. Dundas Grant, **"Some Complications of Nasal Sinus Disease."**

**At 4 p.m.—October 26**, Dr. Newton Pitt, **Clinique (Med.).**

**At 5.15 p.m.**—Sir John Broadbent, Bart., **"The Prognosis and Etiology of Mitral Incompetence."**

**At 4 p.m.—October 27**, Mr. Mayo Collier, **Clinique (Surg.).**

**At 5.15 p.m.**—Dr. Clive Riviere, **"Notes on Tuberculin Treatment."**

**At 4 p.m.—October 28**, Sir Jonathan Hutchinson, **Clinique (Surg.)**

**At 5.15 p.m.**—Dr. F. J. Smith, **"On the Examination of the Person."**

**At 4 p.m.—October 29**, Mr. Hunter Tod, **Clinique (Ear).**

### ST. JOHN'S HOSPITAL FOR DISEASES OF THE SKIN, Leicester Square, W.C.

Chesterfield Lectures.—At the Hospital on Thursday evenings at 6 p.m., each lecture followed by clinical demonstrations :—

**October 28**, **Paratuberculides** (due to Tuberculous Toxins) : I., Macular; II., Papular; III., Pustular; IV., Pigmentary.

### ROYAL SOCIETY OF MEDICINE.

The following are the addresses to be delivered at the meeting of the Odontological Section : on Monday, October 25 : Paper, Mr. A. Hopewell-Smith, "Case of infective disease of the jaws associated with absorption of the teeth"; casual communications, Mr. F. Coleman, (1) Papillomata of the uvula, (2) Exostoses of the mandible, (3) The torus palatinus; Presidential address by Mr. William Hern. Tuesday, October 26, 5.30 p.m., Medical Section. Dr. Robert Maguire : "On oxaluria and the treatment of calcium oxalate deposit from the urine, with a method for the solution of calcium oxalate calculus whilst in the urinary passages." Thursday, October 28, 8.30 p.m., Neurological Section, Presidential address by Professor Sherrington, F.R.S. Friday, October 29, 5.30 p.m., Balneological and Climatological Section : General meeting; ordinary meeting at 6 p.m.—Presidential address by Dr. Leonard Williams; Dr. Eyre, "The Hygiology of Naples." Annual dinner of the Section to follow.

### The Question Box.

**SPECIAL NOTICE.**—Questions of interest affecting the honorary medical staff and hospital administration in all departments are invited. When any point arises we hope our readers will formulate it in a question and so co-operate to make this column of real value and interest. In this way the experience of the best-managed hospitals should be made helpful to the whole hospital world. We shall welcome the contribution of both questions and answers.

**N.B.**—The publication of an answer in this column does not necessarily preclude the publication of subsequent answers to the same question, for a question may involve points which require to be threshed out and fully discussed. Answers, if published, will be paid for at scale rates.

### HOSPITAL FLOORS AND LINOLEUM.

Question 1. What is the most effective and cheapest detergent for removing ordinary stain and varnish from wooden floors?

Question 2. What are the objections to the use of linoleum in hospital wards?

ANSWER : Linoleum keeps the floors damp.

By "Superintendent."

It would be useful to learn of an effective and cheap detergent for removing ordinary stain and varnish applied to wooden floors. The way in which interest in this point arose was as follows :—At a new and fairly large institution over the greater part of the ground floor damp had been found to accumulate under the linoleum laid down there. Linoleum, I believe, has a bad reputation amongst architects and others in this respect. The first measure tried, after all neighbouring steam joints had been seen to, was to cut away a strip of the linoleum along the walls, the boards exposed being stained and varnished. This did not answer, however, and, moreover, a few limited areas of rot appeared. These were cut out and re-planked, some surface draining round one side of the building undertaken, extra ventilators made, and, most important of all, the linoleum taken up. The floors then dried well, but to replace the floor-covering was deemed too risky. The unsightliness of the white parallelograms of bare boards then became very noticeable. The representative of a rather well-known preparation recommended steel-filings and a composition which smelt like benzine; but from a casual mention he made of planing it was anticipated that these would not be of very much practical service in removing the old stain and varnish. Ultimately the impossibility of doing this thoroughly limited our choice of a preparation for the floor to that originally used. With patent compositions, which, too, did not seem to suit deal boards very well, there was a very noticeable "join." But although beeswax is cheap, and the cost of upkeep of stained and varnished floors is light, the initial expense is much greater than with the preparation in question.

[We should be glad to have the experience of a number of our readers on these important points.—Ed. The Hospital.]

### THE PRESS AND THE HOSPITALS.

Question.—What privileges should be accorded to the Press in regard to hospital cases, and especially to accident cases?

ANSWER : The Secretary should judge.

A London View.

By the Hon. Sydney Holland, Chairman of the London and Poplar Hospitals.

I have had some experience in this. At one time, for the sake of advertising the Poplar Hospital, I used to get *Lloyd's* to insert a short account of the most interesting accidents which had occurred during the week and had been taken in. But I was obliged to stop doing so, because I found that a certain class of solicitor watched for these reports and at once pounced down on the unfortunate patient, getting in as a regular visitor, and offered to "take up his case." It is impossible to keep out the determined journalist, but as far as possible the rule ought to be kept that no "interviews" with patients in a hospital should take place except by leave of the Secretary.

ANSWER : Be concise and brief.

A Provincial View.

By "Discens."

The following plan is probably the one which meets with the approval of most hospital boards :

When cases are admitted which are of interest to the general public—cases of gas poisoning, attempted suicide, etc.—the rule is for confirmation of the admission to be made, on application by a Press representative, but no information is given as to the extent of the injury or other information of a strictly professional or private character. The ward report of the case is also subsequently given on application, but is confined to a statement "progressing satisfactorily," "had a fair night," "a little better."

With regard to accident cases very great care is used, first from the point of view of the relatives, who might become unduly alarmed by a vivid newspaper report, and secondly because, with the passing of the Workmen's Compensation and Employers' Liabilities Acts, information might be given which would not be in the interests of either employé or employer, and might furnish a weapon of offence or defence in actions at law which might subsequently be brought.

The barest information is therefore given, and, if questions arise which call for special consideration, the hospital attendants are enjoined to protect themselves from criticism and blame by referring such questions to a higher official.

Experience has shown that this course meets with the approval of the Press, who are usually not unreasonable in their request for news copy.

